# Avascular Necrosis of the Femoral Head in Patients with Antiphospholipid Syndrome: A Case Series

**DOI:** 10.3390/hematolrep17020015

**Published:** 2025-03-21

**Authors:** Paschalis Evangelidis, Eleni Gavriilaki, Nikolaos Kotsiou, Zacharo Ntova, Panagiotis Kalmoukos, Theodosia Papadopoulou, Sofia Chissan, Sofia Vakalopoulou

**Affiliations:** Second Propaedeutic Department of Internal Medicine, Aristotle University of Thessaloniki, Hippocration Hospital, 54642 Thessaloniki, Greece; pascevan@auth.gr (P.E.); nkotsio@auth.gr (N.K.); ntovairo@gmail.com (Z.N.); kalmoukosp@yahoo.gr (P.K.); sissipapth@yahoo.gr (T.P.); sofiachissan@yahoo.com (S.C.); svakalopoulou@yahoo.com (S.V.)

**Keywords:** antiphospholipid syndrome, avascular necrosis, stroke, thrombosis, thrombophilia

## Abstract

**Background/Objectives**: Antiphospholipid syndrome (APS) is a systemic autoimmune disease characterized by thrombosis or obstetric complications and the laboratory detection of antiphospholipid antibodies. Although vascular thrombosis is the main manifestation of the disease, other rarer complications have also been described. Avascular necrosis (AN) is considered a rare manifestation of APS. The aim of our case series is to study patients with APS and AN. **Methods**: A retrospective study was performed on 80 patients diagnosed with APS. **Results**: AN was observed in 3 patients out of 80 diagnosed with APS. AN of the femoral head was observed in all cases. Case (1): A 54-year-old woman presented due to multiple ischemic infarctions in the brain, as detected in magnetic resonance imaging of the brain, Raynaud’s phenomenon, and AN of the femoral head. In laboratory testing, a prolongation of activated partial thromboplastin time was recorded. A heterozygous mutation was also found in the gene MTHFR C677T, and the patients was positive for lupus anticoagulant (LA). The patient was given clopidogrel and acenocoumarol. Case (2): A 52-year-old man was diagnosed with APS, based on the clinical presentation (stroke) and positivity for LA and anti-β2GPI (anti-β2 glycoprotein I antibody). In his medical history, episodes of vertigo and an episode of AN of the femoral head 2 years ago were described. Case (3): A woman aged 43 years presented due to AN of the femoral head. Due to suspected APS, immunological testing was performed, and positivity for LA and IgM anticardiolipin antibodies was detected. She was treated with acenocoumarol. **Conclusions**: AN is a rare clinical manifestation of APS, which may precede the diagnosis of APS for many years.

## 1. Introduction

Antiphospholipid syndrome (APS) is a systemic autoimmune disorder characterized by thrombotic events or obstetric complications and the presence of antiphospholipid antibodies [1]. APS can occur as an isolated clinical entity (primary APS) or might be secondary to another autoimmune disorder, such as systemic lupus erythematosus (SLE) [2]. It is estimated that the prevalence of APS is 40–50 cases per 100,000 individuals, while patients with APS experience higher mortality rates compared to the general population [3]. For many years, the Sydney-revised Sapporo criteria have been used for the diagnosis of APS. The presence of one of the clinical criteria (vascular thrombosis and/or pregnancy morbidity) and one of the laboratory criteria (lupus anticoagulant [LA], anticardiolipin [aCL] antibody, and anti-β2 glycoprotein I antibody [anti-β2GPI]) is essential for diagnosis [4]. The antibodies have to be detected on two or more occasions at intervals of at least 12 weeks.

Recently, the American College of Rheumatology (ACR) and the European Alliance of Associations for Rheumatology (EULAR) published novel classification criteria for APS, based on both clinical and laboratory parameters [5]. Venous and arterial thromboses constitute the major clinical manifestations of the syndrome. At the same time, microvascular thrombosis might also be observed and present as livedo racemosa, livedoid vasculopathy lesions, APS nephropathy, and pulmonary hemorrhage [5]. Other clinical criteria for the diagnosis of APS include obstetric complications, the thickening or vegetation of cardiac valves, and thrombocytopenia. The laboratory diagnosis of APS is based on the identification of persistent positivity for antiphospholipid antibodies (aPL) LA, IgG/IgM aCL, and IgG/IgM aβ2GPI [6]. Laboratory testing for APS should be considered for patients with recurrent thromboembolic events, thrombosis in unusual sites, unprovoked thrombosis in patients younger than 50 years old, or thrombosis and a disproportionately mild environmental provoking factor [7]. Moreover, microvascular thrombotic events should raise awareness of APS, while a personalized approach for every patient is crucial.

Catastrophic APS (cAPS) is the most severe form of APS, affecting a very small proportion of patients with APS, characterized by the formulation of thrombi in small vessels and multi-organ failure [8]. Complement system activation is implicated in the pathogenesis of cAPS, as has been shown by functional and genetic data [9,10]. Moreover, other uncommon presentations of APS include Addison’s syndrome, chorea, transverse myelitis, Budd–Chiari syndrome, acute mesenteric vein thrombosis, and avascular necrosis (AN) of the bone [11,12,13,14]. Low-dose aspirin (LDA), vitamin K antagonists (VKAs), hydroxychloroquine, and prednisone are used for the treatment of patients with thrombotic and obstetric APS [15]. The management of patients with cAPS is more complex, and a combination of glucocorticoids, heparin, plasma exchange, b-cell depletion agents, and complement inhibition has been recommended [15,16,17].

AN is a rare presentation of APS, affecting mainly the femoral head [18]. Furthermore, inherited prothrombotic abnormalities, hypofibrinolysis traits, increased blood viscosity, and platelet activity have been reported as prevalent in patients with AN, especially in refractory cases [19,20]. Patients with osteonecrosis of the hip are often asymptomatic at early stages, while hip and groin pain are the most common clinical manifestations at later stages. Other symptoms include stiffness and difficulties in gait. Magnetic resonance imaging (MRI) has up to 100% sensitivity for the diagnosis of AN of the femoral head [21]. Microvascular thrombosis, endothelial dysfunction, and platelet activation might have a role in the pathogenesis of this APS manifestation [19]. More data regarding AN as a manifestation of APS are essential. For this purpose, in this study, we aim to describe the epidemiology, clinical characteristics, and treatment of patients with APS who presented with AN.

## 2. Materials and Methods

Adult patients diagnosed with APS between 1 January 2009 and 1 June 2024 were enrolled in this study. The diagnosis of APS was made according to the Sydney-revised Sapporo criteria, which are summarized in Supplementary Table S1 [4]. This study was approved by the Hippocration Hospital Ethics Committee (Greece 585/25.7.2023). The informed consent of all patients was provided.

The following data were collected retrospectively from each patient’s medical records: age, gender, coexistence of other autoimmune diseases, arterial and venous thrombosis, obstetric history, livedo reticularis, livedoid vasculopathy lesions, aPL nephropathy, pulmonary hemorrhage, cardiac valve damage, thrombocytopenia, cardiovascular comorbidities, AN, laboratory findings, aPL tests, antibody testing for other autoimmune disorders, and treatments. Arterial and venous events were diagnosed in all patients with imaging methods. In cardiovascular comorbidities, obesity, current tobacco smoking, arterial hypertension, dyslipidemia, and diabetes mellitus were included. The diagnosis of AN was made based on both the clinical findings and MRI. The APL tests conducted were the following: LA, IgG/IgM aCL, and IgG/IgM GPI. Additionally, the persistent presence of aPL for at least 12 weeks was essential, as mentioned above, for diagnosis. The detection of aCL and aβ2GPI was performed with ELISA kits (EUROIMMUN, Luebeck, Germany). LA was measured according to the recommendations of ISTH, using diluted Russel’s viper venom time [22].

## 3. Results

Of the 80 patients with APS, AN was observed in 3 patients. AN of the femoral head was observed in all cases.

### 3.1. Patient 1

A 54-year-old woman presented to our outpatient department due to cognitive impairment and Raynaud’s phenomenon. MRI of the brain was performed, and multiple ischemic infarctions were detected. Three months prior, she developed AN of the femoral head, which was diagnosed with MRI. Otherwise, her medical and family history was free of relevant comorbidities. Due to the clinical suspicion of APS, full clinical and laboratory examination, aPL tests, and genetic tests for thrombophilia were performed. The prolongation of activated partial thromboplastin time (aPTT) (49,4 s) was recorded. The patient was also persistently positive for LA (first testing: 54.7; second testing: 55.3). Based on these findings, the diagnosis of APS was established. Moreover, a heterozygous mutation on the C677T methylenetetrahydrofolate reductase (MTHFR) gene, along with hyperhomocysteinemia, was identified. She was treated with acenocoumarol (target international normalized ratio (INR): 2–3) and clopidogrel. However, 1 year after the diagnosis, she developed deep vein thrombosis (DVT) in the right limb. Thus, the acenocoumarol dose was increased (target INR: 3–4).

### 3.2. Patient 2

A 52-year-old man presented with an ischemic stroke. At the initial diagnostic work-up for the stroke, a cause was not identified. Furthermore, the patient developed AN of the femoral head 2 years before, at the age of 50. MRI was used for the diagnosis of AN. Besides vertigo episodes, the rest of his medical and family history was free of relevant comorbidities. After our diagnostic work-up, the patient was diagnosed with APS based on the clinical findings and persistent positivity of LA (first: 69.2; second: 65.3) and IgG anti-β2GPI (first: 41.3 U/mL; second: 37.3 U/mL). Dyslipidemia, a heterozygous mutation on the C677T MTHFR gene, and hyperhomocysteinemia were revealed. Treatment with acenocoumarol (target INR: 2–3), aspirin, and statin was initiated. To date (48-month follow-up), he has not developed any recurrence of thrombotic events.

### 3.3. Patient 3

A woman aged 43 years presented due to AN of the femoral head (diagnosed with MRI) and non-traumatic ecchymoses in the lower limbs. In her medical history, osteoarthritis of the left knee, syringomyelia due to spinal cord injury, and thalamic arteriovenous malformation were described. Due to suspected APS, immunological testing was performed, and positivity for LA (first: 67.2; second: 60.3) and IgM aCL antibodies (first: 41.2 U/mL; second: 40 U/mL) was detected. We have to underline that this patient did not meet either previous or current APS criteria. She was treated with acenocoumarol (target INR: 2–3). To date (1 year post diagnosis), she has not developed any other complications. In Table 1, the clinical characteristics and treatment outcomes of the patients are summarized.

## 4. Discussion

In our real-world cohort, it was shown that AN of the femoral head is an uncommon presentation of APS. Moreover, we report that AN might be the first clinical manifestation of APS; thus, high clinical suspicion for APS is essential in patients who develop AN. In some cases, AN can be the unique manifestation of APS, or it can be combined with thrombotic complications (such as in patients 1 and 2). APS might coexist with inherited thrombophilic factors [23]. In our study, two out of the three patients had heterozygous mutations in gene C677T MTHFR. However, this polymorphism is not considered a risk factor for arterial or venous thrombosis [24]. More data regarding the role of inherited thrombophilia factors in patients with APS are crucial. Additionally, future research should focus on anticoagulation efficacy based on antibody types and titers in patients with APS.

In a retrospective multicenter study of 1000 patients with APS, the incidence of bone AN was 2.4% [25]. Similarly, in a cohort of 252 Chinese individuals with APS, AN was observed in 4 of them (1.6%) [26]. Carvalho et al. studied 66 patients with APS and showed that AN developed in 3 patients, a similar low incidence to that described in our case series [27]. Moreover, thrombocytopenia and previous glucocorticoid use were recognized as risk factors for AN. Thus, AN is a rare complication of APS. In some cases, differential diagnosis between APS-induced thrombosis and corticosteroid-induced AN is difficult, while in some cases, both of these factors might be implicated. MRI has been used to identify asymptomatic patients with APS and AN, with an incidence of up to 20%, while livedo reticularis is more common in this group of patients [18]. The validity of APS severity scores, such as the global APS score (GAPSS), in the prediction of AN development and risk factors for AN in patients with APS should be investigated in future studies with a large number of participants [28].

In our study, AN was observed in patients with APS, i.e., any other underlying autoimmune disorder. Zonana-Nacach et al. described a case of AN in a 34-year-old male patient also with primary APS [29]. The diagnosis of bone AN in some cases is not easy, and it has to be differentially diagnosed from other clinical entities, such as osteomyelitis [30]. Min Jung and colleagues reported the case of a patient with cAPS who developed multiple osteonecrotic lesions in the spinal cord [31]. In two of our patients, AN was the first manifestation of APS, while Crome et al. described the case of a 73-year-old man who developed AN of the femoral head after rapidly developing digital ischemia and thrombosis of the superior mesenteric artery [32]. Furthermore, besides the femoral head, cases of rib AN in patients with APS have been described [33]. Bone AN has been described as a more common presentation of APS in patients with concomitant HIV infection [34]. Mundo et al. described the case of a liver transplantation recipient who developed APS after the transplantation, and AN of various bones just after the withdrawal of therapy with corticosteroids [35].

Other causes of AN, among others, include acute pancreatitis, steroid and bisphosphonate use, Gaucher’s disease, SLE, and sickle cell disease [36]. The development of AN in patients with immune thrombocytopenia has also been associated with the presence of aPL, along with the chronic use of corticosteroids [37]. Cecchi et al. described the case of a patient with secondary APS (SLE) who developed DVT; thus, the decision to administer an anticoagulant was made, and while being on a Vitamin K antagonist, he presented with multifocal AN [38]. Saleh and colleagues found in their study that in patients with SLE, the presence of aPL and the administration of corticosteroids were associated with the development of AN [39]. Similarly, in the retrospective study of Doğan et al., daily steroid usage was a predictor of AN development in patients with SLE [40]. However, Mok et al. failed to show a correlation between aPL and AN development in patients with SLE. In the recently published study of Hu et al., it was shown that the levels of LA were significantly higher in SLE patients with symptomatic AN in comparison with those who were asymptomatic (*p* = 0.024) [41]. Moreover, they found that the T helper/T suppressor cell ratio was elevated in the symptomatic AN group (*p* = 0.021) [41]. In a meta-analysis of 22 studies including a total of 3054 patients with SLE, an association between aPL and AN development was not identified [42]. Otomo et al. developed the antiphospholipid score (aPL-S), which was found to be efficient in predicting the development of thrombotic events in patients with APS [43]. This score has been reported as a novel risk factor for femoral head AN in patients with SLE [44]. Extensive multicenter studies and investigation into AN etiology in APS are essential, and examining the role of immunity, endothelial dysfunction, and microvascular inflammation are crucial.

Some limitations should be recognized in the current study. Firstly, it was a retrospective study, with a small number of study participants. Thus, statistical analysis was not possible to perform. Moreover, it was a single-center analysis, based on one hematology department. Prospective design and multicenter collaboration would be helpful to overcome these limitations. Finally, we have to underline that regarding the third patient, despite the strong suspicion of APS, the Sydney Classification criteria for APS diagnosis were not met.

## 5. Conclusions

Based on the findings of our study, AN of the femoral head might be an uncommon manifestation of APS but can be presented earlier than the development of other APS complications. Moreover, patients who develop AN that cannot be attributed to another cause should be investigated for APS. More studies, with a larger number of participants, are crucial to better understanding the epidemiology, risk factors, pathogenesis, and outcomes of APS patients with AN. However, given the rarity of this clinical entity, multicenter collaboration aimed at the collection of real-world data can be helpful in this regard. Additionally, research should also focus on the exploration of potential associations between APS-specific biomarkers and bone microvascular pathology.

## Figures and Tables

**Table 1:** Clinical characteristics and treatment outcomes of patients with AN.

Gender	Age	Clinical Manifestations	Corticosteroid Use	Platelet Count (×10^9^ Cells per L) at Diagnosis	aPL	Other Prepositioning Factors for Thrombosis	Treatment	Events During Follow-Up
Female	54	Cognitive impairment (multiple ischemic infracts in the brain), Raynaud phenomenon, Femoral head AN	Νο	222	LA	Heterozygous mutation in gene C677T MTHFR, Hyperhomocysteinemia	Clopidogrel, Acenocoumarol	1 year from diagnosis: DVT of right lower extremity
Male	52	Stroke, History of vertigo, AN of femoral head	Νο	385	LA, IgG anti-β2GPI	Heterozygous mutation in gene C677T MTHFR, Hyperhomocysteinemia	Aspirin Acenocoumarol	-
Female	43	AN of femoral head	Νο	287	LA, IgM aCL	-	Acenocoumarol	-

AN—avascular necrosis; aPL—antiphospholipid antibodies; LA—lupus anticoagulant; MTHFR—methylenetetrahydrofolate reductase; aCL—anticardiolipin antibody; aβ2GPI—anti-β2 glycoprotein I antibody; DVT—deep vein thrombosis.

## Data Availability

The raw data supporting the conclusions of this article will be made available by the authors on reasonable request.

## Supplementary Materials

The following supporting information can be downloaded at: https://www.mdpi.com/article/10.3390/hematolrep17020015/s1.
