# Supplementary material for: Avascular Necrosis of the Femoral Head in Patients with Antiphospholipid Syndrome: A Case Series
Source: Hematol Rep. 2025 Mar 21;17(2):15. doi: 10.3390/hematolrep17020015 (PMC11932201; doi:10.3390/hematolrep17020015)
Supplement: Supplementary file 1 [file hematolrep-17-00015-s001.zip › hematolrep-3379202-supplementary.pdf]

Summary of revised diagnostic criteria for antiphospholipid syndrome [1].

Diagnosis of antiphospholipid syndrome is made when at least one of the clinical criteria and one of the laboratory criteria are present:
Clinical criteria<br>1. Vascular thrombosis<br>2. Pregnancy morbidity
Laboratory criteria
1. Lupus anticoagulant present in plasma, on two or more occasions at least 12 weeks apart<br>2. Anticardiolipin antibody of IgG and/or IgM isotype in serum or plasma, present in medium or high titer, present on two or more occasions, at least 12 weeks apart<br>3. Anti-b2 glycoprotein-I antibody of IgG and/or IgM isotype in serum or plasma, present on two or more occasions, at least 12 weeks apart

References

[1] MIYAKIS, S.; LOCKSHIN, M.D.; ATSUMI, T.; BRANCH, D.W.; BREY, R.L.; CERVERA, R.; DERKSEN, R.H.W.M.; DE GROOT, P.G.; KOIKE, T.; MERONI, P.L.; et al. International Consensus Statement on an Update of the Classification Criteria for Definite Antiphospholipid Syndrome (APS). Journal of Thrombosis and Haemostasis 2006, 4, 295–306, doi:10.1111/j.1538-7836.2006.01753.x.
